# It Ain’t Necessarily So: Ludwig Boltzmann’s Darwinian Notion of Entropy

**DOI:** 10.3390/e26030238

**Published:** 2024-03-08

**Authors:** Steven Gimbel

**Affiliations:** Department of Philosophy, Gettysburg College, Gettysburg, PA 17325, USA; sgimbel@gettysburg.edu

**Keywords:** Ludwig Boltzmann, entropy, Charles Darwin, evolution, model

## Abstract

Ludwig Boltzmann’s move in his seminal paper of 1877, introducing a statistical understanding of entropy, was a watershed moment in the history of physics. The work not only introduced quantization and provided a new understanding of entropy, it challenged the understanding of what a law of nature could be. Traditionally, nomological necessity, that is, specifying the way in which a system must develop, was considered an essential element of proposed physical laws. Yet, here was a new understanding of the Second Law of Thermodynamics that no longer possessed this property. While it was a new direction in physics, in other important scientific discourses of that time—specifically Huttonian geology and Darwinian evolution, similar approaches were taken in which a system’s development followed principles, but did so in a way that both provided a direction of time and allowed for non-deterministic, though rule-based, time evolution. Boltzmann referred to both of these theories, especially the work of Darwin, frequently. The possibility that Darwin influenced Boltzmann’s thought in physics can be seen as being supported by Boltzmann’s later writings.

## 1. Introduction

*The things that you are liable*

*To read in the Bible,*

*It ain’t necessarily so.*

—Ira Gershwin

There is no 10,000 kg sphere of pure gold anywhere in the universe. But that fact’s universality does not make it into a law of nature because while there does not happen to be such a sphere, there could be. Physical laws seem to require a special sort of necessity, what philosophers of science term “nomological necessity”, that tells us what must or cannot happen [1]. Ludwig Boltzmann’s formulation of the Second Law of Thermodynamics [2] was contentious in part because it challenged the traditional understanding of the nature of that necessity in physical law.

Contemporaries like James Clerk Maxwell, Josef Lochschmidt, and Ernst Zermelo objected to Boltzmann’s approach in order to save the traditional account of natural law. If Boltzmann’s proposal clashes with the commitment to traditional necessity, they held, then the proposal should be jettisoned. Boltzmann, on the other hand, opted to revise our understanding of what should be expected from physical laws.

What accounts for Boltzmann’s willingness to be so philosophically radical? The reason may lay in a combination of two factors: (1) Boltzmann’s brand of realism—he was an “entity realist”, believing that atoms do exist, but not a “nomological realist”, believing our best scientific theories merely provide a *Bild*, a useful picture that should not be seen as literally true; and, (2) Boltzmann may have employed Darwinian evolution to provide a *Bild* to understand the microscopic world in which random changes are responsible for the time evolution of a system that is time-irreversible in practice, but not in principle.

Thermodynamic entropy famously emerges from Sadi Carnot’s work on steam engines, the machines that powered the industrial revolution. They ran on coal, which had to be mined. Those coal mines not only provided fuel, but also unearthed the geological strata that led to modern geology. Within the layers of rock were fossils, often of animals different from their modern counterparts and in locations where the animals did not seem to belong. This set the stage for new biological theories of speciation, and ultimately, Darwinian natural selection. 

Huttonian geology and Darwinian evolution are both scientific theories that differentiate temporal directions without the necessity traditionally required in physics, and both were big news in the scientific community when Boltzmann was working on entropy. Geology and biology thus may have provided models for Boltzmann’s thinking about his statistical picture of thermodynamics.

While Boltzmann’s overture is no smoking gun, that is, there is no direct reference to Darwin in Boltzmann’s works in which he develops his understanding of entropy, there are plenty of places both during and after his seminal pieces on statistical mechanics where Boltzmann does not only refer to, but actively employs, Darwin’s thought for a range of purposes. Based upon them, a circumstantial case can be made that Boltzmann used biological evolution as a *Bild* through which to understand the time evolution of thermodynamic systems.

## 2. Maxwell’s Models and Boltzmann’s *Bilder*

Maxwell and Boltzmann held complicated relations to scientific realism, the view that our current best theories reflect reality. Maxwell was famous for his mechanical models, physical analogies that were useful heuristics, but never intended to describe an underlying reality [3]. Playing a similar role in Boltzmann’s understanding of the scientific method was his notion of *Bild*, that is, pictures or models used to make sense of systems, but not necessarily to provide accurate accounts. Indeed, he held that scientists should pursue a range of these models, as each may be fruitful in a different way. 

Yet, while both employed explicitly anti-realist approaches in doing science, both also harbored realist ambitions. If a notion employed in the models developed by scientists, say, of molecules, was sufficiently successful in accounting for a wide enough swath of phenomena without glaring anomalies, then we would have warrant for considering that notion to refer to an actual component of the universe. In both holding most scientific work to be of mere instrumental value, while still allowing that sufficient predictive and explanatory success provided metaphysical license, Henk de Regt [4] calls Maxwell and Boltzmann “flexible realists”.

For Maxwell, absolute truth was restricted to the Divine [5]; only God could know the truths of the universe with certainty. Humans could, at best, develop a rough intuitive sense of the way the world worked, and this was aided by our ability to construct mental metaphors, models that frame the system under investigation in terms of an analogy to another system we understood better. “By a physical analogy I mean that partial similarity between the laws of one science and those of another which makes each of them illustrate the other (quoted in [6] (p. 208))”. Maxwell (and many who followed him) dedicated great effort to constructing cognitively and actually building mechanical models of abstract entities like the magnetic field and the luminferous aether.

As those models became able to account for increasingly greater numbers of observable phenomena, and to predict new ones that had not been previously suspected, the question of the realistic interpretation of the models naturally emerged.

“The question of the reality of analogies in nature derives most of its interest from its application to the opinion, that all phenomena of nature, being varieties of motion, can only differ in complexity, and therefore the only way of studying nature, is to master the fundamental laws of motion first, and then examine what kind of complication of these laws must be studied in order to obtain true views of the universe. If this theory be true, we must look for indications of these fundamental laws throughout the whole range of science, and not least among those remarkable products of organic life, the results of cerebration (commonly called ‘thinking’). In this case, of course, the resemblances between the laws of different classes of phenomena should hardly be called analogies, as they are only transformed identities (quoted in [5] (p. 76))”.

Maxwell thus argues that when a model is sufficiently successful, we cannot but begin to see it as being actually descriptive of the underlying reality. We should never forget that it is a model and not take the model to be a full and complete description, but we must make some limited inference in our grasping of things as they really are.

Boltzmann was deeply influenced by Maxwell’s physics, but also his epistemology. Enticed by the success of Maxwell’s method of theorizing, he, too, hews to a scientific methodology that employs a sort of model at its heart. But where those models were almost exclusively mechanical for Maxwell, Boltzmann moves to a notion that was reflective of what was happening in Austrian culture at the time. 

The notion of “*Bild*” was very much in the air around Boltzmann [7]. On the one hand, it is the term that was used for a photograph, a new technology that led to a wave of philosophical conversation in the culture around reality and representation. Are the properties of the photograph, for example, absolute truths of the world? Is it perspectival or absolutely objective? Could it be used as evidence in a court? In science? What can be inferred about the subject of the photograph from the image itself and with what degree of certainty?

At the same time, it was also the root of the term “*bildung*”, which referred to the process of self-creation through education and culture. In a class-conscious society, as the urban centers of the late Austro-Hungarian Dynasty were, there was a deep connection of the notion of “*Bild*” to what one should believe as a result of a process of discovery. It connoted a relation of the proper orientation of self to the social world.

These senses are embedded in Boltzmann’s Austrian twist on Maxwell’s notion of a mechanical model, turning it into more abstract notion of a scientific *Bild.* While Boltzmann uses the term in a multiplicity of ways [7], it is central to his approach to scientific methodology. A *Bild* is a model that includes elements beyond the observable, mentally developed cognitive constructs. These additional theoretical elements from the mind of the theorist create an explanatory construct which can be used not only to visualize what a system might look like that gives rise to the observed phenomena in the way a Maxwellian mechanical model does, but also to suggest future phenomena that might be accounted for as well. 

In “On the Development of the Methods of Theoretical Physics in Recent Times”, Boltzmann points to Wilhelm Weber’s electro-magnetic theory which, although later discredited by Maxwell’s theory, nonetheless suggested the hitherto undiscovered Hall effect [8]. Because even a *Bild* like Weber’s that turned out not to work beyond the phenomenon it was designed to account for and yet could have such progressive elements, and because a *Bild* should be thought of as a heuristic tool and not an accurate description of the underlying reality, there is an advantage in having theoreticians create a multiplicity of different *Bilder*. In “On the Fundamental Principles and Equations of Mechanics” [9], Boltzmann writes, “If in this way we have grasped the task of thought in general and of science in particular, we obtain conclusions that are at first sight striking. We shall call an idea about nature false if it misrepresents certain facts or if there are obviously simpler ideas that represent these facts more clearly and especially if the idea contradicts generally confirmed laws of thought; however, it is still possible to have theories that correctly represent a large number of facts but are incorrect in other aspects, so that they have a certain relative truth. Indeed, it is even possible that we can construct a system of pictures of experience [*Bildern der Erscheinungen*] in several different ways (pp. 105–106)”.

This multiplicity of distinct models from the same initial set of observations should be seen metaphorically along the lines of mutations in a Darwinian context. Different alterations will have potentially different advantages in terms of the “selection pressures” that are generated by additional experimental discoveries. Indeed, Rosa et al. [10] try to develop a Darwinian epistemology along this line. As such, we see in Boltzmann, as with Maxwell, that a sufficiently successful model should be taken to provide us with some sense that we are developing a picture that is in some limited way representative of the underlying reality.

Maxwell and Boltzmann both allow inferences from sufficiently successful models. The nature of that inference is what is called “entity realism”. If there is an ineliminable element of a model that shows itself to be explanatory and predictive in a wide enough range of situations without significant failure, then there is reason to think that there is a correlate in the real system to that part of the model. In other words, models that are widely applicable without anomaly can give us reason to believe in something we cannot directly observe.

However, this realism does not extend to the model as a whole. While successful models can give us warrant for belief with respect to the furniture of the world, we must always remember that this is a model. Hence, the theory itself will always be at best an approximation, a mere analogy. The parts may point to real things, but the laws are not actual universal truths. While Maxwell and Boltzmann accepted a sort of entity-realism, they both rejected nomological realism, the view that our best current scientific theories tell us how the underlying reality actually operates. 

## 3. The Reality of Molecules

This shared methodological approach led to a temporary disagreement between Maxwell and Boltzmann concerning an inference to the reality of molecules.

Michael Faraday was famously untrained in mathematics and thereby utilized mental pictures as analogies in working out his advances in electricity and magnetism. Maxwell, who was mathematically masterful, translated Faraday’s insights into equations, much to Faraday’s delight [5]. Maxwell continued to use Faraday’s approach of mechanical models prolifically, developing a range of intricate physical analogies. He treated electricity as an incompressible fluid and the ether as a set of cogs. 

But perhaps most importantly, in a series of papers beginning in 1860 with “Illustrations of the Dynamical Theory of Gases” [11], Maxwell constructed mechanical models of gases, treating them as collections of particles that began as impenetrable spheres that interacted only by contact but which became decreasingly idealized as he went on. With each new element added to the mechanical model, more empirical thermodynamic phenomena could be derived.

Maxwell was under no illusion that he was proving anything, but rather saw himself as providing evidence in favor of the kinetic theory of heat [6]. This evidence was the result of our ability to account for an increasing number of observable phenomena and regularities and suggested that this progress would likely continue with the further development of the molecular model. Maxwell famously wrote [11] “If the properties of such a system of bodies [as he assumed] are found to correspond to those of gases, an important physical analogy will be established, which may lead to more accurate knowledge of the properties of matter (p. 377)”.

The success of Maxwell’s research program impressed Boltzmann, who himself began to contribute to it. While both were thoroughly committed to the mechanical theory of heat, in 1871 Maxwell was more reticent to attribute reality to the molecules as a result of an anomaly neither could account for: specific heat. 

The ratio of specific heat at constant volume to specific heat at constant pressure could be experimentally determined. Maxwell’s initial attempt with his simplified picture of molecules failed. This was unsurprising, but when he made the model more realistic, accounting for rotational and translational energies, even while considering the molecules to be polyatomic, Maxwell still could not solve the problem. Boltzmann followed with his attempt, but to no avail [4].

Their inability to account for the correct measured values of specific heats led Maxwell to withhold a realist understanding of the work, referring to it as “the greatest difficulty the molecular theory has yet encountered (quoted in [4] (p. 212))”. Boltzmann contended that the overall progress of the research program justified a realistic interpretation of atoms, where the problem made Maxwell pull back.

Ultimately, Boltzmann did solve the problem by creating the “dumbbell model” of diatomic molecules, which allowed for the rotational and translational motion, but because of the bond connecting the atoms, eliminated one degree of freedom. This changed the theoretical predictions in a way that allowed it to match the measured values. At that point, Maxwell, Boltzmann, and most others—with notable exceptions like Ernst Mach and Henri Poincaré—accepted the existence of atoms as having been demonstrated. His atomic *Bild* gave warrant for an assertion of the reality of its central entity. As Boltzmann would later write, “[C]ontemporary atomism provides a perfectly accurate picture [*vollkommen zutreffendes Bild*] of all mechanical phenomena and, because of the self-contained nature of this field, there is little expectation that we will discover phenomena that do not fit in the frame of this picture [*Rahmen des Bildes*] [12] (p. 150)”.

## 4. How the H Did Boltzmann Decide to Move to a Statistical Law?

Boltzmann accepted the reality of atoms as he worked on the problem of specific heats, also focusing on making sense of other aspects of macroscopic thermodynamics in terms of the mechanical model, specifically, the concept of entropy. How could the macroscopic quantity governing the conversion of energy to work in engines be understood in the microscopic context? There was a macroscopic principle, the Second Law of Thermodynamics, that needed accounting for in terms of Boltzmann’s *Bild*.

Starting in 1872 with his paper “Further Studies on the Thermal Equilibrium of Gas Molecules” [13], Boltzmann sought to model entropy on molecular systems governed by Newtonian mechanics and thereby develop a notion of microscopic entropy that would mirror the macroscopic notion by increasing in systems not in equilibrium and remain constant for those that were. The 1872 paper proposes a theorem involving the quantity that he would term H, which is the negation of entropy. 

Boltzmann followed with a series of papers working on this problem, leading up to the 1877 paper “On the Relationship between the Second Fundamental Theorem of the Mechanical Theory of Heat and Probability Calculations Regarding the Conditions for Thermal Equilibrium” [2], in which he derives what is known as the Boltzmann distribution. In fact, he derives it twice in the paper: once using discrete mathematical means and again employing the continuous means of differential equations. The former, found in Section I of the paper, begins with the “chunking” that Boltzmann included, dividing the molecules into velocity classes and assuming every molecule in that class to have the same velocity, allowing him to deal with a finite number of velocity-classes. (This move, most famously, influenced Max Planck with its idea of quantification which led to his solution of the blackbody radiation problem). As Kim Sharp and Franz Matschinky, in their introduction to their translation of [2], note, “This assumption does not correspond to any realistic model, but it is easier to handle mathematically (p. 1976)”. In Section II of the paper, Boltzmann then repeats the process, but with continuous energy distributions.

This redundancy might seem curious, but as Nadine de Courtenay [14] argues, “Boltzmann was one of the first physicists to recognize that mathematical language was not an inconsequential means of expressing physical processes (p. 50)”. 

Ernst Mach’s insistence on differential equations in physics, for example, was not an innocent choice, Boltzmann argues, but begs the question in favor of his anti-atomism. Mach’s positivism, his epistemological view that we should only believe that which is observable, led him to reject the existence of atoms as well as other in principle unobservable concepts like Isaac Newton’s absolute space. Mach insisted that we replace the sorts of explanations in science that smuggle in unobservable entities and simply see differential equations as the last word, a method that is metaphysically empty, but scientifically full. But this embrace of differential equations as the language of all physics was not philosophically neutral. Mach may have claimed to be trying to rid physics of metaphysical baggage, but he was actually stacking the philosophical deck by choosing to work only in smooth mathematical universes in making his models of the world [14]. Constraining physical laws to the form of differential equations invisibly imported an anti-atomistic metaphysic. Mathematical language is not mere metaphysically neutral formalism, according to Boltzmann, and Mach’s use made him the metaphysician he railed against.

In choosing to work in both discrete and continuous languages, Boltzmann in [2] was dong two things. First, he was playing it metaphysically honestly, showing that his result was not dependent on a particular picture of the underlying world. This is not to say that he was not committed to atomism. 

He was, but in showing that the result was not dependent upon discrete or continuous foundations, he had a second goal: to show that the physics was capable of being a bridge to connect the macroscopic world, in which the microscopic could be treated as if it were smooth, and the microscopic world, which could not. As such, the behavior of the resulting notion of entropy, and the statistical tools he would build around it, would provide a picture that holds true in both frames of reference. It worked for the macroscopic world that was seen as if it was continuous and for the microscopic world that had to be treated as discrete. By having a single treatment that is invariant under the change from discrete to smooth, the work had to be seen as bridging the intellectual chasm between the macroscopic thermodynamic and the microscopic atomistic.

But there was an aspect of his result that undermined this bridge. The resulting view of entropy was statistical. What began in the 1872 paper as an epistemic probability, a statistical generalization resulting from our inability to account for the multitude of atoms in a small amount of a gas, turned into a fully stochastic approach by 1877 in which the entropy was no longer tied to properties of the individual atoms in the gas, but rather now became a measure of the accessibility of abstract ensemble states. “Boltzmann’s explanation was to consider the increase in entropy as a result of a statistical process, involving the calculation of probabilities, and not as a result of a dynamic process described only by mechanics [10]”. 

Boltzmann did not give up his commitment to the atomic hypothesis, but moved his thinking from the Maxwellian approach of deriving the macroscopic directly from mechanical aspects of the microscopic to a higher-level picture. In shifting the object of the law from standardly physical quantities like duration and velocity to properties of the constructed phase space, Boltzmann was radically revising how to think of the system and how to understand the macroscopic law governing entropy. The Second Law of Thermodynamics was no longer deterministic, but a statistical generalization that made what we see not necessary, but highly likely. That move, of course, generated serious objections. 

## 5. Defending Necessity

So, according to Boltzmann’s approach, entropy tends to increase, thereby distinguishing future from past, but does not always necessarily increase, and that probability is intrinsic to the system, not a mere result of our ignorance. Critics, notably Maxwell, Josef Lochschmidt, and Ernst Zermelo, objected to different parts of the position. Maxwell to the nature of the probability, Lochschmidt to the lack of time-reversibility, and Zermelo to the lack of necessity.

Maxwell did not object to the statistical move per se, but tried to show that the system is not stochastic. The uncertainty involved remains of the epistemological sort. His eponymous demon is a tiny intelligence in control of a valve. The demon is capable of determining the velocity of all molecules of a gas and capable of opening or shutting the vale at will. By choosing to only open the valve for high-velocity molecules, the demon thus becomes capable of sorting the molecules and thereby creating increased order, that is, of decreasing entropy. 

Entropy thereby does not necessarily increase, but our statistical sense that it generally does is a result of our not being demonic, that is, of our having less cognitive capacity than the fanciful being. Because its ability to alter the amount of entropy in the system is accomplished through the intelligent processing of information, then, the appeal to probability is epistemic and not metaphysical. In other words, the statical nature of the Second Law would be a result of our ignorance, not a feature of the world itself [7] (p. 1278).

Josepf Lochschmidt, was a dear and close friend of Boltzmann. They both accepted the existence of atoms and the supposition that they were governed by Newtonian mechanics. But if this is true, Lochschmidt contended, then the properties of the laws governing the small would have to be the same as those governing the large since the ensemble is nothing more than a collection of necessarily determinable states. The rules governing the parts—which are deterministic and time-reversible—should not differ from those governing the whole. 

Yet, Boltzmann’s statistical approach creates an asymmetry between future and past. This, Lochschmidt argues, is problematic. “[I]n any system, the entire course of events becomes retrograde if at a certain moment the velocities of all its elements is reversed (quoted in [15] (p. 200))”. Given that Newtonian mechanics is time-reversible, reflecting the velocities of all molecules would follow Newton’s laws and decrease entropy, but this reversed system is as much a model of Newton law as the non-reversed one. As Flamm points out of the reflection, “This procedure is equivalent to time reversal”, that is, the backward running film of the universe decreases entropy, but still fits the underlying mechanics Boltzmann uses.

It should be noted that this was also a line that was used against Darwin’s theory of evolution. Critic William Hopkins [16] wrote, “a phenomenon is properly said to be explained, more or less perfectly, when it can be proved to be the necessary consequent of preceding phenomena, or more especially, when it can be clearly referred to some recognized cause; and any theory which enables us to be to do this may be said in a precise and logical sense, to explain the phenomenon in question. But Mr. Darwin’s theory can explain nothing in this sense, because it cannot possibly assign any necessary relation between the phenomena and the causes to which it refers them (p. 267)”.

Zermelo’s objection took a different route. If the entropy of a system is based upon the distribution of positions and velocities, and these obey Newtonian principles, then given enough time, the system will eventually find itself back in the original orientation. But, since there was an increase in entropy moving away from the initial state, there would have to have been a decrease in order for the original state to recur.

The Second Law of Thermodynamics is a purported law of nature. Laws of nature are more than mere rules of thumb. They say what must happen. Making a law of nature into a statistical generalization is to undermine it as a law of nature. In physics, during that period, Hermann Bondi [17] contends, “it was widely thought that the perfect predictability of Newton’s solar system ‘clockwork’ was what any physical science, nay what any human endeavor should aim to achieve (p. 159)”. Boltzmann’s understanding of entropy denied it and therefore, to many, it seemed not to be good science. As Bondi reported Ernest Rutherford as having said, “If you get a statistical answer, you asked the wrong question”. 

Boltzmann wrestled with all three of these concerns, acknowledging that they were, indeed, concerns that needed to be taken seriously. And yet, he did not waver from his approach. Formulating different versions of responses to them, he always stayed true to his approach. He was willing in the move from the micro- to the macroscopic to accept a deeply stochastic picture of nature which differentiated future from past, but which did not possess the sort of necessity that had been considered an essential aspect of physical law. What accounts for this digging in of his intellectual heels in the face of strong objections to the different aspects of his view from people he respected? 

One possibility is another successful *Bild*, another cognitive framework that allowed him to make sense of this unusual world in a way that made sense to him. That heuristic could have been provided by Darwin’s theory of natural selection.

## 6. Darwin in Germany and Austria around Boltzmann

Darwin’s *Origin of Species* first appeared in German translation in April 1860, translated by Germany’s most prominent paleontologist, Heinrich Georg Bronn, whose own research had been proceeding parallel to Darwin’s [18]. However, Bronn passed away two years after the translation’s publication. As a result, Darwinism in Germany required someone else to act as its spokesperson. Stepping into this role was the young, handsome, and charismatic Ernst Haeckel who famously spoke to the German Society of Naturalists and Physicians at Stettin in September of 1863, and with an address of historical significance, the twenty-nine year old zoologist launched the era of Darwinism in Germany. “With the translator Bronn”, he told the assembled crowd, “I see in Darwin’s direction the only possible way to come close to understanding the great law of development that controls the whole organic world [18] (p. 157)”.

The approach that Haeckel develops, and which becomes greatly influential in the German-speaking world during Boltzmann’s time, however, is less Darwinian and more in line with that of Jean Baptiste Lamarcke. We see this sort of Lamarckian understanding of Darwin in the writings of Hermann von Helmholtz [19]. Boltzmann studied with Helmholtz in Berlin in 1871, just before Boltzmann’s initial work on the H theorem, and we find in Helmholtz’s personal notebook from that period (notes which he used to give his lectures) passages like the following:

“Recapitulation of Darwin’s hypotheses: The law of heredity demonstrates a range of variations in all classes of organisms although the fewest differences arise among species in the wild. What remains to be determined is the limits of this change. This is where the following comes into consideration: that a much greater influence is to be expected from natural breeding than from artificial selection. The hypothesis of an independent origin of each species becomes incredibly unlikely as a result of (a) the homology among different species (b) Metamerism within the same species (c) Paleontological developments, and (d) the geographical affinity of like organisms to live together”.

[20]

The reference here to “metamorism” shows the ways in which Helmholtz saw Darwin as connected to his own research program on color perception and thereby would have likely been a topic Helmholtz would have been thinking about during the period when Boltzmann was in contact with him.

Boltzmann left Berlin for a position back in Vienna which he occupied until 1876, just before the publication of his statistical approach to entropy. At that time, his colleague was Ernst Mach, who had been installed in the newly created chair in “the history and philosophy of the inductive sciences”. In this position, which Boltzmann himself would later occupy, Mach became a public spokesperson for his philosophical position of positivism. While the scientific and epistemological disagreements between Mach and Boltzmann are extremely well-trod ground, one point of agreement between them was their mutual enthusiasm for Darwinism (see [21]). Indeed, Mach asserts that it must have its imprint on all of human thought. For example, in his inaugural address “On Transformation and Adaptation in Scientific Thought” [22] for his position at Prague (the post he held before moving to Vienna), Mach writes, that “[K]nowledge, too, is a product of organic nature. And although ideas, as such, do not comport themselves in all respects like independent organic individuals, and although violent comparisons should be avoided, still, if Darwin reasoned rightly, the general imprint of evolution and transformation must be noticeable in ideas also (quoted in (pp. 217–218))”.

While we can trace Boltzmann’s public and published references to Darwin at least as far back as 1886, nine years after his transformative approach to entropy, it is clear that Boltzmann would have been surrounded by discussion of Darwin’s theory from the wider culture and from important intellectual figures with whom he was interacting at the time he was working on this new understanding of the Second Law.

## 7. Darwin in Boltzmann

There is no smoking gun. We do not have Boltzmann in a correspondence or a footnote citing Darwin’s influence on his thought in developing his statistical understanding of the Second Law of Thermodynamics. However, what we do have is a range of testimony from Boltzmann about a wide range of other ways that Darwin influenced his thinking at the time and afterward, some closer and others farther from this topic. We can also find analogues in the treatment and evidence of natural selection that mirror the arguments raised against Boltzmann’s approach to the Second Law of Thermodynamics, and in the analogous situations, the concerns cease to be serious concerns. Putting this together, we may not be able to demonstrate that Boltzmann was thinking of the time evolution of ensembles of atoms along the lines of the adaptations of species in ecosystems, but it would fit comfortably within the larger confines of how we do know that Boltzmann dealt with other issues in epistemology and science.

Indeed, Darwin occupied a privileged place in the science of this time. In 1886, nine years after the statistical turn and while Boltzmann is working on the argument addressing Loschmidt’s objection in print in his article “Neuer Beweis zweier Sätze über das Wärmegleichgewicht unter mehratomigen Gasmolekülen”, he is called to give a talk to the Austro-Hungarian Imperial Academy on the Second Law of Thermodynamics [23]; he began by reflecting on the centrality of science in general on human progress.

“If we regard the apparatus of experimental natural science as tools for obtaining practical gain, we can certainly not deny its success. Unimagined results have been achieved, things that the fancy of our forebears dreamt in their fairy tales, outdone by the marvels that science in concert with technology has realised before our astonished eyes. By facilitating the traffic of men, things and ideas, it helped to raise and spread civilization in a way that in earlier centuries is paralleled most nearly by the invention of the art of printing. And who is to set a term to the forward stride of the human spirit! The invention of a dirigible airship is hardly more than a question of time. Nevertheless I think that it is not these achievements that will put their stamp on our century: if you ask me for my innermost conviction whether it will one day be called the century of iron, or steam, or electricity, I answer without qualms that it will be named the century of the mechanical view of nature, of Darwin [23] (p. 15)”.

Boltzmann thought Darwin more important than air travel. 

It should not be lost that this is in a paper discussing the notion of entropy and that instead of calling Darwin’s work “the theory of evolution” or “speciation by natural selection”, instead Boltzmann chooses a name that explicitly parallels the phrase he used for the kinetic theory of gases—the mechanical view of heat. Indeed, Boltzmann’s discussion seeks to erase the distinction between the explanatory sciences like physics and the merely descriptive historical sciences. “Since the mighty upswing of geology, physiology and so on, but above all since the general acceptance of the ideas of Darwin, these sciences boldly undertake to explain the forms of minerals and of organic life [23] (p. 16)”. With the “mighty upswing” of geology and the great success of Darwin, there were examples of a mechanical approach to the world in which the development of a scientific system is the result of accidental, not deterministic factors, a fact that was not lost on Boltzmann. 

Boltzmann not only admired the theory of evolution; he was clearly reading Darwin closely. Boltzmann reflects on the most profound questions of humanity “whence do we come, whither shall we go” and asserts that “essential and undeniable progress” has been made in “the present century, thanks to most careful studies and comparative experiments on the breeding of pigeons and other domestic animals, on the coloring of flying and swimming animals, by means of researches into the striking similarity of harmless to poisonous animals, through arduous comparisons of the shape of flowers with that of the insects that fertilize them (p. 14)”. This is a list, almost chapter by chapter, of the evidence that Darwin sets out for his theory in *The Origin of Species*. Clearly, Boltzmann not only read the book, but knew its structure and argumentation intimately.

The influence of Darwin can be seen in multiple ways in the thought of Boltzmann: two epistemological, one metaphysical, and two scientific.

### 7.1. Darwin as an Answer to Kantianism

The structure of the human mind was a lively question among physicists of Boltzmann’s time. The advancements of science required a foundation in epistemology. We had to know what knowledge was in order to continue to increase it. The prevailing trend in Continental philosophy in the 19th century moved away from the scientific and toward the romantic. The idealism of Friedrich Hegel ruled supreme in German-speaking philosophy departments. As such, philosophers seemed of little use when facing the challenge of non-Euclidean geometry or the rise of atomism, which posited the existence of the unobservable.

Some, like Boltzmann’s contemporary at Vienna, Mach, took on a thoroughgoing empiricism, arguing that only what could be observed should play a part in our understanding of the world. Diametrically opposed to the *Naturphilosophie*, this meant the elimination of God and other purely speculative metaphysical entities like the spirit. Influenced by the work of Gustav Fechner, Mach [24] contended that we could do away not only with the idea of the soul, but with the notion of mind altogether, replacing it with a purely materialistic psychophysics. This monism stood as the key move in establishing a general stance: “all metaphysical elements are to be eliminated as superfluous and as destructive of the economy of science (xxxviii)”. Having done away with the mind/soul, Mach also sought to cleanse science of Newton’s absolute space and absolute time, atoms, and any other theoretical construct that was not reducible to sense perceptions.

Others did not take Mach’s positivist route, but sought the last major philosophical figure who did take science seriously, which sparked a rebirth of Kantianism in Germany. Immanuel Kant argues in his *Critique of Pure Reason* [25] that certain mathematical and scientific notions, such as Euclidean geometry and Newtonian mechanics, are what he terms “synthetic a priori”. The logical distinction between analytic and synthetic propositions distinguishes between those like “bachelors are unmarried” and “bachelors are slobs”, wherein analytic sentences like the former have a predicate that is contained in the meaning of the subject, whereas synthetic sentences like the latter have a predicate that is not a part of the meaning of the subject. The a priori/a posteriori distinction is epistemological in separating those propositions that could be known without perception from those that require observation. 

Before Kant, it was supposed that all analytic propositions are a priori and all synthetic propositions are a posteriori, but Kant argues that there is a subset of propositions that are synthetic a priori, i.e., that we know without experience, but which are not merely definitional. Consider the Euclidean axioms. We know that you can draw a circle of any size around any point, but this truth is not merely one that is arrived at through unpacking the notions of circle and point. It is a synthetic proposition in possessing content beyond the definition of the words making it up.

Kant’s question, then, is “How are a priori synthetic judgements possible?” His answer is that they are an innate part of the structure of the human mind. They function as the categories by which the mind takes the raw manifold of perception, such as the blur of colors taken in by the eyes, and out of them constructs complex perceptions. The sensory organs provide the content of what we observe, but the necessary categories of the mind provide the form and when the activity of the mind acts upon the raw data fed in from the senses, the result is the observations we make of the world around us.

Because they are innate, they are universal. All humans begin with the same conceptual foundations. Observation can add to it, but all people start with the same set of categories to construct the world. And since these are the structural elements implicit within our observations, no possible experience could contradict them. They are apodictic, viz., necessarily true and in principle unfalsifiable. Because these are the ideas that build the world out of our perceptions, no perception could possibly falsify it, since it built those perceptions.

A number of major figures who championed a revived version of the Kantian synthetic a priori as the basis for their justification of scientific truths were scientists Boltzmann held in the greatest respect. Heinrich Hertz, for example, writes in the prefatory note to his book *The Principles of Mechanics: Presented in a New Form* [26]:

“The subject-matter of the first book is completely independent of experience. All the assertions made are a priori judgments in Kant’s sense. They are based upon the laws of the internal intuition of, and upon the logical forms followed by, the person who makes the assertions; with his external experience they have no other connection than these intuitions and forms may have (p. 45)”.

Indeed, Hertz and Boltzmann had a lively correspondence with Hertz contending with Kant that the laws of thought must be synthetic a priori [27].

Boltzmann’s response in [28] is to counter this brand of neo-Kantianism, which he thinks is so absurd as to be a joke, with Darwin.

“What then will be the position of the so-called laws of thought in logic? Well, in light of Darwin’s theory they will be nothing else but inherited habits of thought. Men have gradually become accustomed to fix and combine the words through which they communicate and which they rehearse in silence when they think, as well as the memory pictures of those words and everything in the way of internal ideas used for the denoting of things, in such a manner as to enable them always to intervene in the world of phenomena in the way intended, and in inducing others to do likewise, that is to communicate with them. These inventions are greatly promoted by storing and suitable ordering of memory pictures and by learning and practicing speech, and this promotion is the criterion of truth. This method for putting together and silently rehearsing mental images as well as spoken words became increasingly perfect and has passed into heredity in such a way that fixed laws of thought have developed…One can call these laws of thought a priori because through many thousands of years of our species’ experience they have become innate to the individual, but it seems to be no more than a logical howler of Kant’s to infer their infallibility in all cases…According to Darwin’s theory this howler is perfectly explicable. Only what is certain has become hereditary; what was incorrect has been dropped. In this way these laws of thought acquired such a semblance of infallibility that even experience was believed to be answerable to their judgement. Since they were called a priori, it was concluded that everything a priori was infallible (pp. 194–195)”.

Boltzmann is adopting an early version of the view that would later be famously held by Noam Chomsky, that a result of evolution is an inherent linguistic faculty that is innate and results in a particular intrinsic grammar beneath all human language-use [29]. This explains the correct elements of the Kantian synthetic a priori, Boltzmann contends, without committing us to the flawed apodictic aspect.

Boltzmann’s epistemological approach was pragmatic, seeing the forming of beliefs based on observations as evidence as a successful adaptation of our ancestors. The human mind is an artifact of evolution and so, therefore, must the structures by which it determines what we ought to believe based upon what it is fed through the sense organs. Darwin, instead of Kant, forms the basis for his understanding of why the human mind is capable of scientific theorizing and testing.

What is most important in the current context, though, and should be emphasized, is that the move Boltzmann is making here epistemologically is to undermine the apodictic nature of Kantian synthetic a priori truths, that is, the necessity of these propositions, preferring instead a view in which they are the result of a Darwinian evolutionary process by which they arise accidentally through a historical process. This is very much homeomorphic to the move Boltzmann makes in 1877 with respect to the Second Law of Thermodynamics, taking it from a proposition whose necessity must be asserted to the result of probabilistic historical processes.

### 7.2. Darwin as a Metaphor for the Scientific Method

Darwin provides not merely the basis on which we should understand why humans are capable of science, but also provides a way of understanding the means by which scientific results become rationally accepted by the scientific community. Boltzmann writes in [30] “[N]o theory is absolutely true, and equally hardly any absolutely false either, but that each must gradually be perfected, as organisms must according to Darwin’s theory. By being strongly attacked, a theory can gradually shed inappropriate elements while the appropriate residue remains (p. 153)”.

Evolution is not only a theory, but a theory that provides an image for how theories emerge, survive, and grow. Rosa et al. in [10], expand upon this metaphorically Darwinian approach in their article “Constructivism and Realism in Boltzmann’s Thermodynamic Atomism”.

### 7.3. Darwin as Support for the Materialist Worldview

Boltzmann’s atomistic worldview not only provided him with a picture of the microscopic workings of thermodynamic systems, but provided a thoroughgoing ontology (that is, a catalogue of everything that exists in reality). Heat had been considered a substance from Aristotle to the advocates of phlogiston theory. The mechanical theory of heat allowed for the simplification of our catalogue of things in the universe, and Boltzmann saw the mechanical theory of heredity as doing the same. 

In the German-speaking world of the 19th century, one of the most important scientists connected with evolution was Haeckel, most remembered for his aphorism “Ontogeny recapitulates phylogeny”. Coming out of the *Naturphilosophie* movement which leaned heavily on research in embryology and morphology paired with a robust religious metaphysic, Haeckel developed an explicitly evolutionary theory, albeit one that was more in line with that of Lamarck than Darwin’s approach. But Haeckel [31] added a German philosophical twist to his view, placing Geist (spirit or soul) at the heart of the biological.

“No reproach is more frequently made against the science of to-day, especially against its most hopeful branch, the study of development, than that it degrades living Nature to the level of a soulless mechanism, banishes from the world the ideal, and kills all the poetry of existence. We believe that our unprejudiced, comparative, genetic study of soul-life gives the lie to that unjust accusation. For if our uniform or monistic conception of Nature is rightly founded, all living matter has a soul, and that most wondrous of all natural phenomena that we usually designate by the word spirit or soul is a general property of living things. Far other than believing in a crude, soulless material, after the manner of our adversaries, we must rather suppose that the primal elements of soul-life, the simple forms of sensibility, pleasure, pain, the simple forms of motion, attraction, and repulsion, are in all living matter, in all protoplasm. But the grades of the up-building and composition of this soul vary in different living beings, and lead us gradually upwards from the quiescent cellsoul through a long series of ascending steps to the conscious and rational soul of man (p. 173)”.

Contrary to the more mechanical picture of Darwin, Haeckel is explicit in his insertion of the metaphysical into the material. Life is a combination of matter and soul, body and spirit.

Boltzmann rejected the metaphysical dualism inherent in the position, asserting that all phenomena from astronomy to psychology could be accounted for in terms of the behavior of the material constituents of the world. The biggest challenge for this sort of metaphysical materialism, of course, is human consciousness. But Boltzmann [12] sees Darwin as having given us the tools for that.

“The brain we view as the apparatus or organ for producing word pictures [Bilder], an organ which because of the pictures’ great utility for the preservation of the species has, comfortably with Darwin’s theory, developed in man to a degree of particular perfection, just as the neck of the giraffe and the bill of the stork have developed to an unusual length (p. 69)”.

The human mind is no more mysterious than any other of a range of biological curiosities. Humans are just animals and our intelligence is just one more example of a notable adaptation. From [32], “From a Darwinian point of view we can grasp furthermore what is the relation of animal instinct to human intellect. The more perfect an animal, the more it shows incipient traces of intellect alongside instinct (p. 138)”.

### 7.4. A Darwinian Explanation of the Development of Photosynthesis, Mind, and Life Itself

Life requires decreasing entropy, as such an essential element of life is the collection of free energy for the purpose of doing the work needed to maintain life. Using his understanding of radiative entropy, Boltzmann was able to make sense of the process according to Englebert Broda [21]. Boltzmann contends that plant photosynthesis arose and developed during a Darwinian struggle for improved supply of free energy:

“The dependence of plant life on light had been discovered in London by Jan Ingen-Housz, a Dutchman mostly living in Vienna, in 1779. However, Ingen-Housz did not know why exactly light is needed. Indeed he could not know it as conservation of energy was unknown in his time, and so the need for a particular source of energy did not seem to exist. The second step had been taken by the discoverer of energy conservation (First Law of Thermodynamics), Julius Robert Mayer, in 1845. He wrote “The plants absorb a force, light, and produce a force: chemical difference”. However, in his ignorance of the Second Law Mayer could not make a distinction between useful and useless forms of energy. This distinction, in respect to photosynthesis, was left to the physicist who understood the Second Law better than anybody else, and who explained it in atomistic terms, to Ludwig Boltzmann. In 1884 he had introduced the notion of the entropy of radiation (p. 62)”.

This sort of combination of his approach to thermodynamics and Darwin’s natural selection, Broda shows, is not limited to photosynthesis, but also gives an account of the arising of other bioenergetic processes such as fermentation and respiration. Indeed, Broda points to passages in Boltzmann’s later lectures in which he makes this form of argument for the arising of consciousness and life itself.

### 7.5. Darwin as a Model for Thermodynamics

Again, while we do not have Boltzmann asserting the connection between Darwinian evolution and the time evolution of thermodynamic systems as a part of his thinking in the 1877 move to a statistical understanding of entropy, we do have instances of Boltzmann publicly connecting the two after the fact. Boltzmann employs Darwin’s thought as a *Bild*, a heuristic model, for the development of statistical mechanics. There is significant textual evidence that the two are connected in Boltzmann’s thought after the development of his statistical notion of entropy, which allows for an inference that Darwinian evolution may have served as a structural model for Boltzmann in developing his statistical understanding of thermodynamics. 

In [12], Boltzmann connects mental phenomena, which he understands as material interactions that are the result of human evolutionary history, with the physical phenomena of electricity and, more importantly, for this point, heat. “Mental phenomena may well be much more remote from material ones than thermal or electric from purely mechanical ones, but to say that the two former are qualitatively while the latter three are only quantitatively different seems to me mere prejudice (72)”. While Boltzmann used the term “mechanical” in different ways in various contexts, here, he is clearly meaning something along the lines of being governed by the laws of mechanics (as opposed to Maxwell’s equations); as such, he is not creating a mechanical picture of mind in the sense that the mind is a result of Newton’s laws of motion, but he is creating a mechanical theory of mind which does not require any sort of non-material soul.

It is mere prejudice, a fallacy to be eliminated, to distinguish between mental and thermal systems. They are not identical, but of the same sort, Boltzmann contends. As such, the type of arguments that support evolutionary outcomes would not be of a different epistemic species from the sort from those we give in making arguments about thermodynamics.

Indeed, we see in his “Reply to a Lecture on Happiness Given by Professor Ostwald” [33], an evolutionary discussion that maps very closely to a discussion of molecules in a gas. “As regards the concept of happiness, I derive it from Darwin’s theory. Whether the in the course of aeons the first protoplasm developed ‘by chance’ in the damp mud of the vast waters on the Earth, whether egg cells, spores, or some other germs in the form of dust or embedded in meteorites once reached Earth from outer space, is here a matter of indifference. More highly developed organisms will hardly have fallen from the skies. To begin with there were thus only very simple individuals, simple cells or particles of protoplasm. Constant motion, so-called Brownian molecular motion, happens with all small particles as is well-known; growth by absorption of similar constituents and subsequent multiplication by division is likely explicable by purely mechanical means. It is equally understandable that these rapid motions were influenced and modified by their surroundings. Particles in which the change occurred in such a way that on average (by preference) they moved to regions where there were better materials to absorb (food), were better able to grow and propagate so as soon to overrun all the others (176)”.

The approach to evolution reduces the generally macro-level interaction of members of species with their environment, giving rise to selection pressures which affect the relative success of mutations, and places it in a microscopic framework in which atomic-level interactions, like Brownian motion, are in play and driven “by chance”. Framing the process stochastically while speaking of the average speed of small particles in rapid motion is directly parallel to the sort of calculations Boltzmann was undertaking in understanding the time evolution of gases with notions like the mean free path. The convergence of vocabularies and concerns between the two is not only striking, it is clearly intentional. Boltzmann is explicitly connecting evolution with thermodynamics. 

It should be noted that the Professor Ostwald to whom Boltzmann is responding is Wilhelm Ostwald, a Nobel laureate who was one of the last major opponents of atomism, preferring a system based on energy as a foundational concept. From his [34], “What we hear originates in work done on the ear drum and the middle ear by vibrations of the air. What we see is only radiant energy which does chemical work on the retina that is perceived as light. When we touch a solid body we experience mechanical work performed during the compression of our fingertips…From this standpoint, the totality of nature appears as a series of spatially and temporally changing energies, of which we obtain knowledge in proportion as they impinge upon the body and especially upon the sense organs which fashioned for the reception of the appropriate energies (159)”. 

Ostwald was seeking to do away with particles, making energy the primary metaphysical constituent of the universe. In his evolutionary example, he is taking organisms—a clear instance of things—and intuitively elevating them so that the evolutionary example rhetorically supports his atomism.

It should be noted that Boltzmann uses in his example here, in the influence of Brownian motion. Ironically, it was Jean Perrin’s experimental work on Brownian motion that led Ostwald to finally relent and reject his energetics theory for atomism. Perrin’s experiments verified the theoretical work of Albert Einstein [35]. Boltzmann could not have known at the time that Brownian motion would have this effect on Ostwald or that Einstein would explain it in terms of atomic interaction several years later. Indeed, as John Blackmore [36] persuasively argues, it is unlikely that Boltzmann was aware of Einstein’s work on his matter.

We see a similar move on the part of Boltzmann in [32]: “We must mention also that most splendid mechanical theory in the field of biology, namely the doctrine of Darwin. This undertakes to explain the whole multiplicity of plants and the animal kingdom from the purely mechanical principle of heredity, which like all mechanical principles of genesis remains of course obscure (p. 132)”.

Note the phrasing “that most splendid mechanical theory in the field of biology”. Boltzmann is creating a class of mechanical theories, of which the mechanical theory of heat is the most obvious example, but then including with a celebratory nod a member of the group in biology. 

But the most important element comes a couple of pages later when Boltzmann highlights an unexpected element of the evolutionary process: the expected unexpected, that is, the instances in which random mutations give rise to unfit organisms. “It is well-known that Darwin’s theory explains by no means merely the appropriate character of human and animal bodily organs, but also gives an account of why often inappropriate and rudimentary organs or even errors of organization could and must occur (136)”.

Darwinian evolution is driven by random mutations (although they did not understand the genetic force driving it at the time). That randomness will not always give rise to more fit organisms, but will be expected to create dead ends at an expected statistical rate. Yes, we generally focus on the developments that drive the process forward, but given that it is a random process there will also be expected cases of the undesirable and indeed the unexpected. 

Evolution can run backward. The eye can develop, but then if a subpopulation takes to inhabiting caves, selection pressures could undo what eons of selection processes had constructed. It is the general case, but not absolutely necessary, that members of species become more complex to fit their environment.

This is an instance of Darwinian evolution enacting its own version of Zermelo’s objection. It is perfectly possible that a strange set of circumstances could take a species and do and then undo all of the changes. Evolution is a time-oriented theory, yet there is a miniscule possibility that it will return that on which it works to its original stage. It is highly unlikely, but theoretically conceivable. That does not mean that evolution has not been working. It is just an unusual possibility given the statistical nature of the process.

But, if we accept this likelihood in the biological sense, Boltzmann seems to be saying, why would we have any problem with the analogue in the thermodynamic case? Clearly, we should not, and if Boltzmann was using Darwinian evolution, the mechanical theory of inheritance, as a *Bild* underlying his construction of statistical mechanics, then we have a perfectly reasonable explanation for why none of the concerns and objections raised would have seemed to be concerning to Boltzmann.

## 8. Conclusions

In 1877, Ludwig Boltzmann made a stunning shift. His new version of the Second Law of Thermodynamics was a statistical regularity, lacking the standard sort of nomological necessity that had been traditionally asserted as an essential property for a law of nature. It was a bold move that was novel in physics, but not in science writ large. Huttonian geology and especially Darwinian evolution, two theories that were tremendously prevalent in scientific discourse at the time, possessed structural similarities. In the decades after his shift in the understanding of entropy, Boltzmann refers to both of these theories.

This is especially true with respect to Darwin’s work, of which Boltzmann was a vocal supporter. Indeed, we see Boltzmann model elements of his approach to the scientific method on Darwin. Evolution plays a central role in his understanding of the acquisition of human knowledge, of the emergence of life and consciousness, and Boltzmann considers ways in which his work on entropy must be a part of biological evolution itself. 

All of this was after 1877, but given Boltzmann’s time in Berlin with Hermann von Helmholtz, whose work bridged the physical and biological and who was thinking and speaking about Darwin, there is reason to believe that Darwin was on Boltzmann’s mind just before his bold new understanding of entropy emerged.

After his view solidified, Boltzmann used Darwinian analogies to explain his work to the public. Boltzmann saw the connection between his approach and Darwin’s to be similar enough to be able to use the latter as a *Bild*, a conceptual picture capable of making the latter more clear to the mind.

As a result of all of this circumstantial evidence, there seems to be warrant for considering the possibility that Darwin helped Boltzmann make the switch that appears in 1877. Again, there is no smoking gun here, but we do have a variety of different sorts of evidence garnered around a hypothesis. The inference based upon that is similar to Darwin’s own approach to argumentation in *The Origin of Species*, an inductive method of inference he learned from his own teacher William Whewell [37], who called such an approach to reasoning “consilience”. 

What strikes the contemporary mind as odd about this claim is that a biological system could be used as a model for a physical system. Going back to August Comte and through the Logical Positivists (who develop in part from the ideas of Philipp Frank, Boltzmann’s own student), the reductionist picture of science has psychology reducing to biology, which is just complicated chemistry, which in turn is nothing but physics. As such, when we look at the origin of ideas in the most basic of the sciences, physics, then surely the only influences are to be found in the discourse among physicists. But recall what Boltzmann himself thought his own era was the age of—the age of Darwin. While it may seem to those who have internalized the reductionist scheme to put the intellectual cart before the scientific horse, Boltzmann’s own epistemology freed the scientist to find an appropriate *Bild* wherever one could. 

If this inference is correct, then, Darwin and Boltzmann become the scientific Porgy and Bess with the reworked Gershwin’s lyric: “The phase space sectors where you’d find a system’s vectors…it ain’t necessarily so”.

## Data Availability

No new data were created or analyzed in this study. Data sharing is not applicable to this article.
